# Wave speed mapping visualizes the cavotricuspid isthmus reconnection area: A case report

**DOI:** 10.1002/ccr3.9548

**Published:** 2024-11-03

**Authors:** Hiroshi Mase, Hiroyoshi Mori, Tatsuya Onuki, Hiroto Sugiyama, Ayumi Omura, Taku Asano, Hiroshi Suzuki

**Affiliations:** ^1^ Division of Cardiology, Department of Medicine Showa University Fujigaoka Hospital Yokohama Japan; ^2^ Division of Cardiology, Department of Medicine Showa University Hospital Yokohama Japan

**Keywords:** ablation, atrial flutter, cavotricuspid isthmus, omnipolar mapping, wave speed mapping

## Abstract

**Key Clinical Message:**

In addition to the reentrant map, the wave speed map can be helpful in accurately identifying the CTI gap during radiofrequency application for atrial flutter(AFL). However, in complex cases involving extensive scarring and multiple low‐velocity local areas, this technique may not be useful.

**Abstract:**

A 73‐year‐old male patient with a history of pulmonary vein isolation and cavotricuspid isthmus ablation underwent a second catheter ablation owing to recurrent atrial flutter (AFL). The AFL was diagnosed as cavotricuspid isthmus‐dependent AFL caused by the reconnection of the previous cavotricuspid isthmus ablation. Wave speed mapping was performed at the same site, and results comprehensively revealed a low‐velocity local area. The AFL was terminated after the first radiofrequency application, and the block line was easily completed. Therefore, this technique could be an adjunctive tool for cavotricuspid isthmus gap identification and minimal radiofrequency application.

## 1 INTRODUCTION

Recently, wave speed mapping has recently been introduced for detecting low‐ or high‐velocity conduction sites with the omnipolar mapping technology using a three‐dimensional mapping system (EnSite™ X EP System; Abbott, Chicago, IL, the USA). Herein, we reported the efficacy of this system in a patient with atrial flutter (AFL) recurrence.

### 1.1 Case history and physical examination

A 73‐year‐old male patient who underwent pulmonary vein isolation and cavotricuspid isthmus (CTI) ablation for persistent atrial fibrillation and AFL exhibited recurrent AFL. The patient had palpitation symptoms and underwent a second ablation.

### 1.2 Investigations, diagnosis, and treatment

**FIGURE 1:**
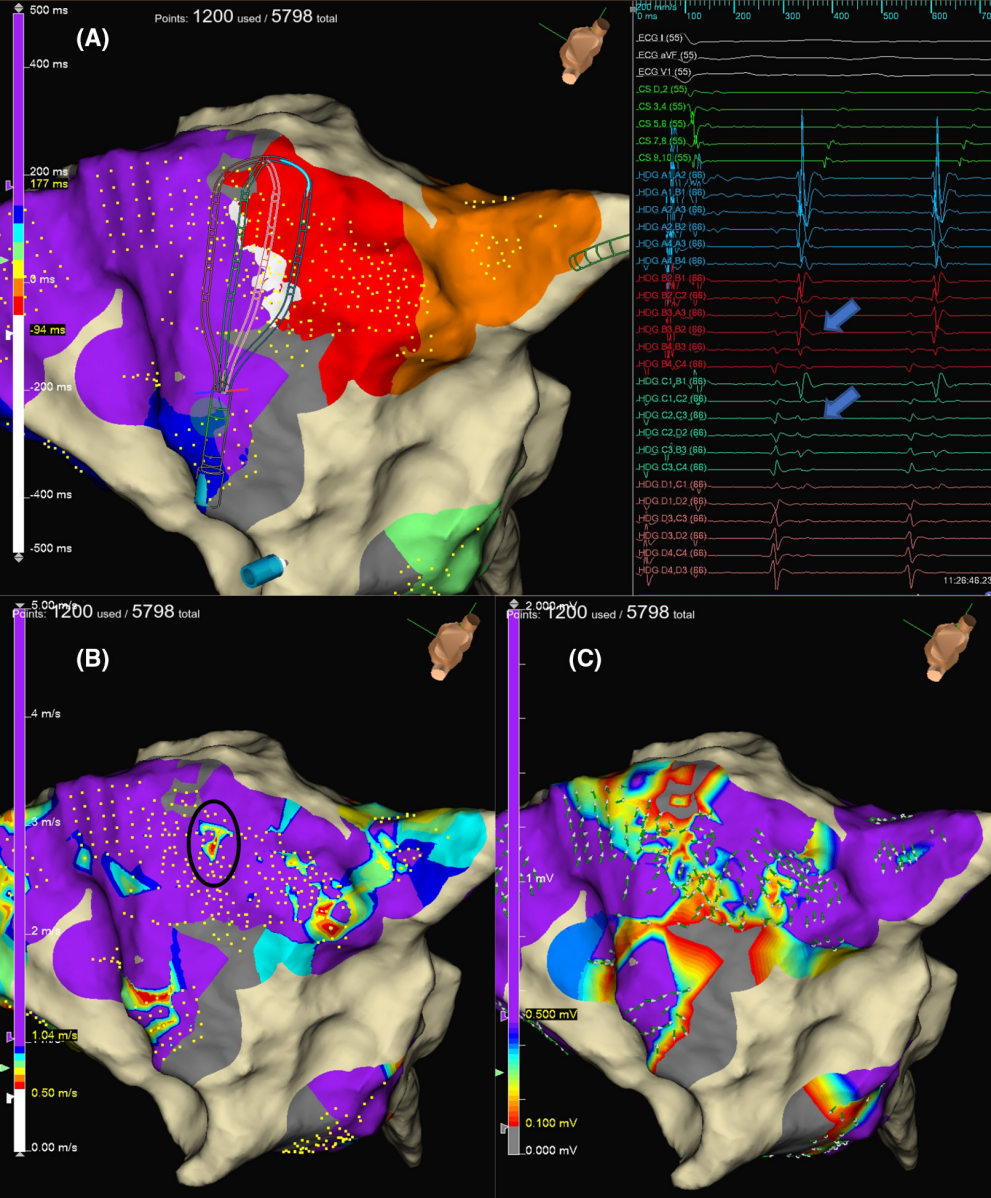
The potentials by a high‐density (HD) grid mapping catheter placed in the cavotricuspid isthmus during atrial flutter (AFL) in the reentrant map. The local potentials consist of short double potentials with no obvious fragmental potentials. (A) The bottom left figure shows a wave speed map (B), and the bottom right figure displays a vector and voltage map. (C) The reentrant map presents reconnection from the inferior vena cava side. In contrast, the wave speed map displayed a low‐velocity local area on the tricuspid annulus side within the black circle, and the AFL was terminated at the first radiofrequency application to the same area. The evident reconduction gap in the vector map could not be visualized. There was no specific voltage decreases in the low‐velocity local area, similar to other areas.

There was no reconnection of all pulmonary vein. Subsequently, we induced AFL with programmed electrical stimulation and mapped AFL using a high‐density (HD) grid mapping catheter to diagnose CTI‐dependent AFL. Entrainment pacing was performed but AFL stopped, then we could not measure the post pacing interval. The potentials by HD grid mapping catheter consisted of short double potentials without obvious fragmental potentials. The voltage, reentrant, vector, and wave speed maps were created in the same area, and a low‐velocity local area was observed near the tricuspid valve (Figure 1). The mean velocity in this area was 0.66 ± 0.13 m/s (with an average value of 5 points at the low‐velocity local area), whereas that in the other area was 2.08 ± 0.94 m/s (with an average value of 82 points at all sites, except for the low‐velocity local area). The voltage on the CTI line was low. Contact force ablation was performed using the TactiCath™ Contact Force Ablation Catheter, Sensor Enabled™ (Abbott, St. Paul, MN, the USA), wherein the power was limited to 30 W and the contact force was maintained at 10–20 g.

### 1.3 Outcome and follow‐up

**FIGURE 2:**
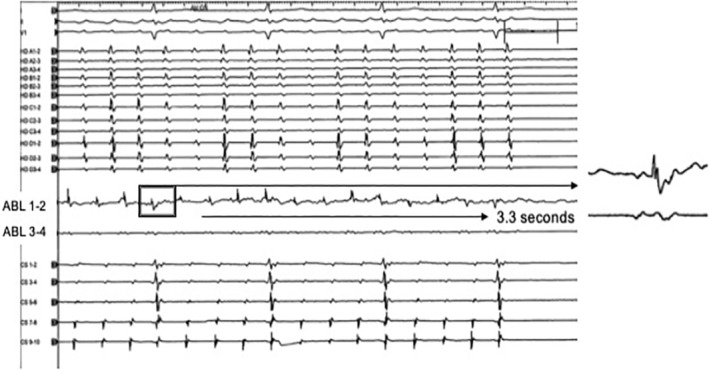
The local potential before radiofrequency application in a low‐velocity local area. The distal potential of the ablation catheter consists of a short double potential. However, no obvious continuous fragmental potential. Atrial flutter (AFL) was terminated at the first radiofrequency application.

We ablated the low‐velocity local area, and the CTI‐dependent AFL was terminated 3.3 s after the first radiofrequency application; furthermore, the CTI block line was easily completed (Figure 2). The patient has had no recurrence to date.

## 2 DISCUSSION

In this case, wave speed mapping was used in CTI ablation to visualize the reconnection gap as the low‐velocity local area, resulting in a successful block line. The low‐velocity conduction areas in the CTI are substrates of the reentrant circuit.^1^, ^2^ A previous study that measured conduction velocity in a patient with recurrent CTI revealed a local conduction velocity of 1.8 ± 0.7 m/s on the lesion line and 0.3 ± 0.1 m/s on the reconnection gap.^3^ The conduction velocity values measured in our case were similar and seems to be consistent. Wave speed mapping can extract sites with different local conduction velocities, including the accessory pathway.^4^ However, its usefulness remains unclear. A recent study used omnipolar vectors and reported a CTI ablation strategy that targets regions with a low‐velocity conduction and high‐voltage identification, suggesting that wave speed mapping is useful for CTI ablation.^5^ The omnipolar mapping technology based on a three‐dimensional mapping system (EnSite™ X EP System, Abbott) provides a detailed explanation of how the wave speed is determined using unipolar and bipolar signals, thereby deriving omnipolar signals, directions, and conduction velocities as the wave speed. In addition, similar to that in voltage mapping, conduction velocity in wave speed mapping can be displayed flexibly. We demonstrated that the reconnection gap in previously treated lines can delay local conduction velocities compared with those at other sites; these conduction delays can be visualized using wave speed mapping.

## 3 IN CONCLUSION

In the present case, there was only one CTI gap, which might have accentuated the conduction delay in the wave speed map. This technique may not be useful in patients with extensive scarring and multiple low‐velocity local area identified during a macro‐reentrant tachycardia. However, it can be combined with the previously used the reentrant map to more accurately identify the CTI gap and provide a supplementary tool for minimal radiofrequency application. Hence, the block line can be easily completed.

## AUTHOR CONTRIBUTIONS

**Hiroshi Mase:** Writing – original draft. **Hiroyoshi Mori:** Supervision. **Tatsuya Onuki:** Writing – review and editing. **Hiroto Sugiyama:** Validation. **Ayumi Omura:** Validation. **Taku Asano:** Validation. **Hiroshi Suzuki:** Supervision.

## FUNDING INFORMATION

This study received no funding.

## CONFLICT OF INTEREST STATEMENT

All authors have no conflicts of interest to disclose.

## CONSENT

Written informed consent was obtained from the patient to publish this report in accordance with the journal's patient consent policy.

## Data Availability

The data that support the findings of this study are available on request from the corresponding author. The data are not publicly available due to privacy or ethical restrictions.
